# Effects of the Tobacco Defensin NaD1 Against Susceptible and Resistant Strains of *Candida albicans*

**DOI:** 10.3390/pathogens13121092

**Published:** 2024-12-10

**Authors:** Olga V. Shevchenko, Alexander D. Voropaev, Ivan V. Bogdanov, Tatiana V. Ovchinnikova, Ekaterina I. Finkina

**Affiliations:** 1 M.M. Shemyakin and Yu.A. Ovchinnikov Institute of Bioorganic Chemistry, Russian Academy of Sciences, 117997 Moscow, Russiaovch@ibch.ru (T.V.O.); 2 Moscow Center for Advanced Studies, 123592 Moscow, Russia; 3 G.N. Gabrichevsky Research Institute for Epidemiology and Microbiology, 125212 Moscow, Russia

**Keywords:** plant defensins, tobacco NaD1, *Candida albicans*, candidiasis, resistance, clinical isolates, human β-defensin 2 (HBD2), human cathelicidin LL-37, caspofungin, adherence, biofilms, synergism, membrane-disrupting activity

## Abstract

Today, *Candida albicans* is still the most common cause of both local and life-threatening systemic candidiasis. The spread of resistant fungal strains has resulted in an urgent need to search for new promising antimycotics. Here, we investigated the antifungal action of the tobacco defensin NaD1 against susceptible and resistant to azoles and echinocandins strains of *C. albicans*. We demonstrated that NaD1 was equally effective and fungicidal against all tested strains. The MIC and MFC values were 6.25 and 12.5 µM, respectively. We showed for the first time that NaD1 could act synergistically not only with caspofungin but also with human host defense antimicrobial peptides cathelicidin LL-37 and β-defensin-2 (HBD2) against susceptible and resistant fungal strains. Using flow cytometry, we demonstrated that NaD1 in combinations with LL-37 or HBD2 can reinforce each other by enhancing membrane disruption. Using the Caco-2 cell monolayer model, we demonstrated that NaD1 impaired the adhesion of *C. albicans* cells to the human epithelium. Moreover, NaD1 inhibited the formation of fungal biofilms in Sabouraud broth and less markedly in nutrient-rich RPMI-1640 medium, and enhanced the antibiofilm activity of caspofungin. Thus, we hypothesized that NaD1 might affect the development of candidiasis in vivo, including that caused by resistant fungal strains.

## 1. Introduction

*Candida albicans* is a commensal whose overgrowth is usually suppressed by the human immune system. However, decreased immune defense and increased fungus pathogenicity lead to opportunistic infections. Today, *C. albicans* is a common cause of not only superficial mouth, vagina or skin infections but also life-threatening invasive candidiasis worldwide [1,2].

Many different virulence factors of *C. albicans* contribute to the development of infection. The morphological switch from yeast-like to hyphae forms plays a key role in the development of invasive candidiasis [3]. Hyphae formation leads to the penetration of the fungus into the bloodstream and decreases the capacity of the phagocytic cells to uptake and kill fungal cells [4]. *C. albicans* produces the hyphae-derived peptide toxin candidalysin, which is encoded by the *ECE1* gene, interacts with epithelial cell membranes, forms pore-like structures and causes membrane damage [5]. The adherence of *C. albicans* to human epithelia often leads to the formation of biofilms. The formation of biofilms contributes to fungal resistance to phagocytosis and significantly reduces sensitivity to antifungal drugs [6,7]. ALS and Hwp1 adhesins are the key virulence factors of *C. albicans* [5]. A wide variety of hydrolytic enzymes of *C. albicans* take part in biofilm formation, destroy epithelial cells and hydrolyze secreted host defense factors [8]. Pra1 cleaves the central component of human complement C3 and functions as a fungal immune-escape protein [5].

Despite *C. albicans* infections responding well to treatment, a growing body of evidence has suggested that resistance among clinical isolates of this fungus is rising [1]. The most common form of azole resistance develops due to overexpression or point mutations in the *ERG11* gene encoding lanosterol-14α-demethylase [9]. Gain-of-function mutations in the ergosterol biosynthesis pathway regulator *UPC2* also lead to increased *ERG11* expression [5]. Resistance to polyenes occurs as a result of replacement of cell membrane ergosterol with alternative sterols [10]. Resistance to echinocandins results from point mutations in the *FKS* genes encoding (1,3)-β-D-glucan synthase [11]. Mutations in the mismatch repair gene *MSH2* can also result in echinocandin resistance and in cross-resistance to azoles [5]. Overexpression of transmembrane transporters *CDR1*, *CDR2* and *MDR1*, caused, for example, by gain-of-function mutations in the genes of transcription factors *TAC1* and *MRR1*, facilitates an elimination of antifungal drugs from *C. albicans* cells, making a significant contribution to fungal resistance [5,12]. Multiresistant strains of *C. albicans* pose a tremendous threat due to the failure of drug therapy. This is why the search for new antifungal drugs with various mechanisms of action, exhibiting high activity against not only susceptible but especially resistant fungal strains, is of paramount importance for clinical practice. Plant defensins are considered to be new antifungal agents for candidiasis treatment.

Defensins represent components of plant innate immunity and their main function is believed to be to protect the plant from phytopathogenic fungi [13]. A lot of available data have demonstrated that these antimicrobial peptides (AMPs) can display activity not only towards phytopathogenic fungi but also against fungi that are pathogenic for humans. The compact structure of these peptides is characterized by high stability. Plant defensins have a similar spatial organization but low amino acid homology, which probably leads to the diversity of their targets and mechanisms of antifungal action. Common targets of these peptides are universal or fungal-specific components of the cell membrane [13,14]. It was demonstrated that plant defensins such as DmAMP1 from *Dahlia merckii*, radish RsAFP1 and RsAFP2, rice OsAFP and maize ZmD32, as well as NaD1 from tobacco flowers, have pronounced antifungal activity against *C. albicans* [15]. However, today, little is known about the potency of plant defensins against resistant and multiresistant fungal strains.

The aim of this study was to investigate the antifungal potential of the tobacco defensin NaD1 against susceptible and resistant to azoles and echinocandins strains of *C. albicans*. Firstly, we investigated the antifungal activity of NaD1 against planktonic cells of various fungal strains. Secondly, a search for new effective combinations of NaD1 with other antifungal agents was performed. Conventional antimycotics from different classes, ebselen, which exhibits antifungal properties, and human host defense AMPs were used in these experiments. Thirdly, the impact of NaD1 on the adhesion of *C. albicans* cells to the epithelium was investigated using the Caco-2 cell monolayer model. Finally, the ability of NaD1 to affect fungal biofilm formation was investigated in various environments, and the potential to enhance the antibiofilm effect of caspofungin was evaluated.

## 2. Materials and Methods

### 2.1. Materials

Collection strains of *C. albicans* ATCC 18804 and ATCC 10231, as well as clinical isolates of *C. albicans* v47a3, 1.1, 9.1, 14.1 and 8.2 obtained from patients with human immunodeficiency virus (HIV) infection and provided by the G.N. Gabrichevsky Research Institute for Epidemiology and Microbiology (Moscow, Russia), were kept at −70 °C in 10% non-fat milk with 10% glycerol.

Colorectal adenocarcinoma Caco-2 cells (ATCC HTB-37) were cultured in complete DMEM/F12 (1:1) (Gibco, Waltham, MA, USA) medium containing 10% fetal bovine serum (FBS, Capricorn Scientific, Ebsdorfergrund, Germany) and 1× antibiotic–antimycotic solution (AA, Invitrogen, Waltham, MA, USA) in a humidified CO_2_ incubator (5% CO_2_, 37 °C).

The conventional antimycotics caspofungin (Sigma, St. Louis, MO, USA), micafungin (Macklin, Shanghai, China), anidulafungin (Macklin, China), amphotericin B (Sigma, USA) and voriconazole (Sigma, USA), as well as ebselen (Sigma, St. Louis, USA), which exhibit antifungal activity, were used in this study.

Recombinant tobacco defensin NaD1 (UNIPROT Q8GTM0), as well as human host defense antimicrobial peptides β-defensin 2 (HBD2, UNIPROT O15263) and cathelicidin LL-37 (UNIPROT P49913), were obtained by heterologous expression in *E. coli* cells by using plasmid constructs pET-His8-TrxL-NaD1/LL-37/HBD2, as described previously [16]. The recombinant peptides were purified from clarified cell lysates by metal chelate chromatography, dialysis, acid cleavage of the fusion proteins with cyanogen bromide, repeated metal chelate chromatography and RP-HPLC. Homogeneity and the identity of the recombinant peptide samples were confirmed by MALDI mass spectrometry and CD spectroscopy [16].

### 2.2. Antifungal Activity Assay

*C. albicans* cells in stock were inoculated onto Sabouraud agar plates with 2% glucose and incubated for 24 h at 37 °C. After replating, cells were inoculated in Sabouraud broth and cultured at 37 °C for 2 h. Cell concentration was determined using a LUNA-II cell counter (Logos Biosystems, Dongan-gu Anyang-si, Republic of Korea) and trypan blue (Logos Biosystems, Dongan-gu Anyang-si, Republic of Korea).

Antifungal susceptibility tests were performed by the microdilution method in 96-well flat-bottom microplates using low-salt Sabouraud broth or nutrient-rich medium RPMI-1640 (Capricorn, Germany), mainly as described [17]. Yeast-like cells at a concentration of 4 × 10^4^ cells/mL in Sabouraud broth or double RPMI-1640 were mixed with equal volumes of serial dilutions of NaD1, LL-37 or HBD2 in water. Serial dilutions of antimycotics amphotericin B in water or caspofungin, micafungin, anidulafungin and ebselen in 1% DMSO, as well as voriconazole in 1% methanol, were also used. Control samples without test substances were also tested. The 96-well microplates were incubated at 30 °C for 24 h. Fungal growth was assessed using an inverted microscope (Olympus, Tokyo, Japan) and also by measuring absorbance at 630 nm. MIC_50_ (in the case of voriconazole) and MIC were defined as minimal concentrations of antifungal agent, causing 50 and 100% inhibition of fungal growth. The minimum fungicidal concentration (MFC) was defined as the lowest concentration of antifungal substance at which there was no cell growth after plating on Sabouraud agar with 2% glucose and 20 μg/mL chloramphenicol (Sigma, USA) and incubation at a temperature of 37 °C for 24 h, the contents of the plate wells with concentrations equal to the MIC and higher. Sabouraud broth with the addition of 150 mM NaCl or 1.25 mM MgCl_2_ or 1.25 mM CaCl_2_ or 10% FBS was used to estimate the influence of various salts and serum on the antifungal activity of NaD1. All experiments were performed twice in triplicate. To prevent the adsorption of antifungal substances, the wells of microplates were previously blocked with 0.1% BSA.

To study the morphological switch of different *C. albicans* strains, cells were inoculated in Sabouraud broth or RPMI-1640 and cultured at 37 °C for 4 h after that microscopic analysis was performed.

### 2.3. Checkerboard Antifungal Assay

Serial two-fold dilutions of the mixture of two combinable antifungal substances were mixed with equal volumes of *C. albicans* cells at a concentration of 4 × 10^4^ cells/mL cultured in Sabouraud broth. After incubation of the 96-well microplates at 30 °C for 24 h, fungal growth was assessed, as mentioned above. To assess the combined effect of antifungal substances, the fractional inhibitory concentration index (FICI) was calculated using the formula FICI = ([A])/([MIC_A_]) + ([B])/([MIC_B_]), where [MIC_A_] and [MIC_B_] are the MICs of the substances A and B, respectively, and [A] and [B] are the concentrations of the substances A and B in iso-effective combinations, respectively.

### 2.4. Inhibition of Biofilm Formation

For biofilm formation, 100 µL of *C. albicans* cell suspensions at concentration of 10^6^ cells/mL in Sabouraud broth or RPMI-1640 were entered into the wells after that the plates were incubated at 37 °C for 24 h. Formed biofilms were washed with PBS to remove nonadhered cells, stained with 0.1% crystal violet (Sigma, USA) for 15 min and washed gently with PBS. In the case of *C. albicans* strains incapable of forming biofilms, cell staining was carried out without washing procedures.

To estimate the effects of NaD1, caspofungin and their combination on biofilm formation, *C. albicans* cells in Sabouraud broth or RPMI-1640 at a concentration of 2 × 10^6^ cells/mL were mixed with equal volumes of serial two-fold dilutions of antifungal compounds in corresponding medium [18]. Inhibition of biofilm formation was assessed by measuring the absorbance of biofilms washed by PBS at 630 nm and by using the resazurin-based method. In the final case, resazurin (Sigma, USA) at a final concentration of 0.7 mM in PBS was added to the washed biofilms. Microplates were incubated for 4–6 h and fluorescent resorufin was registered using a 535/595 filter in a PlateReader AF2200 (Eppendorf, Hamburg, Germany). Untreated cells were used as the negative control. All experiments were performed twice in triplicate. BIC_50_ and BIC were defined as the minimal concentrations of antifungal agent causing 50 and 100% inhibition of biofilm formation, respectively. The biofilm inhibitory concentration index (BICI) was calculated as BICI = ([A])/([BIC_A_]) + ([B])/([BIC_B_]), where [BIC_A_] and [BIC_B_] are the BICs of the substances A and B, respectively, and [A] and [B] are the concentrations of the substances A and B in iso-effective combinations, respectively.

### 2.5. Prevention of Adhesion to the Epithelium

The adhesion of different strains of *C. albicans* to the epithelium was studied using a Caco-2 cell monolayer as an in vitro model of the intestinal barrier, similar to that described in a previous study [19]. Caco-2 cells were seeded into 96-well flat-bottom plates in complete DMEM/F12 medium containing 10% FBS and 1× AA solution and incubated in a humidified CO_2_ incubator. Cells were grown over 14 days and medium was changed twice a week. The presence of a cell monolayer was checked using an inverted microscope; after that, the plates were incubated for one more week to ensure complete polarization of Caco-2 cells. The day before the experiment, the culture medium was replaced with fresh complete DMEM/F12.

*C. albicans* cells in stock were inoculated onto Sabouraud agar plates with 2% glucose and incubated for 24 h at 37 °C. After replating, cells were cultured in complete DMEM/F12 at 37 °C for 2 h and were diluted up to the concentration of 10^6^ cells/mL. Then, 50 µL of the resulting cell suspension (5 × 10^4^ cells) and equal volumes of solutions of NaD1, caspofungin or their combinations in complete DMEM/F12 were added to each well. The plates were incubated for 1.5 h at 37 °C in a CO_2_ incubator; after that, the Caco-2 monolayer was washed 2 times with PBS to remove unattached *C. albicans* cells. To separate adherent cells, 10 µL of trypsin/EDTA solution (PanEco, Moscow, Russia) was added to each well. After incubation of microplates for 10 min, 90 µL of complete DMEM/F12 with 10% FBS was added to inhibit trypsin activity. The well contents were scraped and diluted 100-fold with PBS, after which 10 µL aliquots were plated on Sabouraud agar with 2% glucose and chloramphenicol at a concentration of 20 µg/mL. The plates were incubated at 37 °C for 24 h, and cell adhesion was assessed by counting the grown fungal colonies. *C. albicans* cells not treated with antifungal compounds were used as a negative control. This experiment was carried out twice in triplicate. % Adhesion was calculated as ((average number of adhered cells on plate × 100)/5 × 10^4^) × 100%. % Adhesion inhibition was calculated as ((average number of adhered cells in sample − average number of adhered cells in control)/average number of adhered cells in sample) × 100%.

### 2.6. Proteinase Activity

The proteinase activity of clinical isolates of *C. albicans* was studied using bovine serum albumin (BSA, Sigma) agar, mainly as described [20]. The medium contained 2% agar, 0.25% KH_2_PO_4_, 0.02% MgSO_4_ × 7H_2_O, 0.5% NaCl, 0.1% yeast extract, 2% glucose and 0.25% BSA as the sole nitrogen source, pH 4.0 or 7.0, was used. Solutions of BSA and glucose sterilized by filtration were added to the medium, which was autoclaved and cooled to 50 °C. Ten microliters of clinical isolates of *C. albicans* at a concentration of 10^6^ cells/mL in Sabouraud broth were used to inoculate the surface of the BSA agar; after that, the plates were incubated at 37 °C for 4 days. Then, the plates were fixed with 50% methanol in 10% acetic acid, stained with 0.1% amido black in 45% methanol and washed with 30% methanol in 10% acetic acid. The clear zones around colonies were manually measured. The experiment was performed twice, using 4 and 9 technical replicates.

### 2.7. Membrane Permeability Assay Using Flow Cytometry

A membrane permeability assay was performed using the detection of propidium iodide (PI) uptake on a Novocyte 2060R flow cytometer (ACEA Biosciences Inc., San Diego, CA, USA), equipped with 488 nm blue and 640 nm red lasers [17]. *C. albicans* ATCC 18804 was used for this study at a final concentration of 2 × 10^4^ cells/mL in Sabouraud broth. Yeast-like cells were incubated in shaking conditions at 30 °C for 2 or 20 h at a volume of 4 mL in tubes pre-blocked with 0.1% BSA in the presence of or without NaD1, caspofungin, LL-37 or HBD2 at 0.25× the MIC, 0.5× the MIC, the MIC and 4× the MIC. Different combinations of NaD1 with caspofungin, LL-37 or HBD2, corresponding to the synergistic or additive action of these substances, were also used. Untreated cells and those heat-killed at 99 °C for 15 min were used as controls of live and dead *C. albicans* cells. After incubation, the cells were centrifuged at 1600× *g* for 10 min, resuspended in PBS and stained for 20 min at room temperature in the dark with PI at concentration of 4 μg/mL (Biotium, Fremont, CA, USA). The obtained data were processed using NovoExpress Software v. 1.2.4 (ACEA Biosciences Inc., San Diego, CA, USA).

### 2.8. Hemolytic Assay

The hemolytic activity of NaD1 was studied in 96-well microplates by using fresh human red blood cells (hRBCs), as described [17]. hRBCs in PBS (final concentration of 4% (*v*/*v*)) were mixed with serial two-fold dilutions of NaD1 in PBS (final concentrations from 0.78 to 50 μM) and incubated for 2 or 24 h at 37 °C. Aliquots of the supernatants containing released hemoglobin were transferred to 96-well microplates after intact hRBC sedimentation, and the absorbance at 405 nm was measured. hRBCs in PBS or 0.1% Triton X-100 were used as negative and positive controls, respectively. The percentage of hemolysis was calculated as % hemolysis = ((A_sample_ − A_negative control_)/(A_positive control_ − A_negative control_)) × 100%. The experiment was carried out twice in triplicate. Membrane-active peptide melittin from honeybee venom was used for comparison.

## 3. Results and Discussion

### 3.1. Characteristics of Susceptible and Resistant Strains of C. albicans

In this study of the antifungal properties of the tobacco defensin NaD1, we used sensitive and resistant to azoles collection strains of *C. albicans* ATCC 18804 and ATCC 10231, respectively, as well as resistant to azoles and echinocandins clinical isolates v47a3, 1.1, 9.1, 14.1 and 8.2 of *C. albicans* obtained from patients with human immunodeficiency virus (HIV) infection (Table 1). We have demonstrated that v47a3 is the most sensitive and 8.2 is the most resistant strain to conventional antimycotics (Table 1; Supplementary Materials Table S1). It has previously been shown that some of these clinical isolates are characterized by overexpression of the *ERG11* gene and/or *CDR1*, *CDR2* and *MDR1* transmembrane transporters genes [21] (Supplementary Materials Table S2). In order to further characterize the selected strains, we studied their ability to the morphological transition from a yeast-like to a hyphal form, their adherence to the epithelial monolayer surface and their capacity to form biofilms and produce secreted proteases.

*C. albicans* is a fungus present in both yeast-like and hyphae forms that differ in cell morphology, gene expression, growth dynamics and invasion potential [6]. We revealed that only strain 8.2 changed its morphology and turned into a hyphal form in Sabouraud broth. The switch from a yeast-like to hyphal form in all strains of *C. albicans* used, except clinical isolate v47a3 and susceptible strain ATCC 18804, took place after cultivation in nutrient-rich RPMI-1640 (Table 1; Supplementary Materials Figure S1).

**Figure 1:**
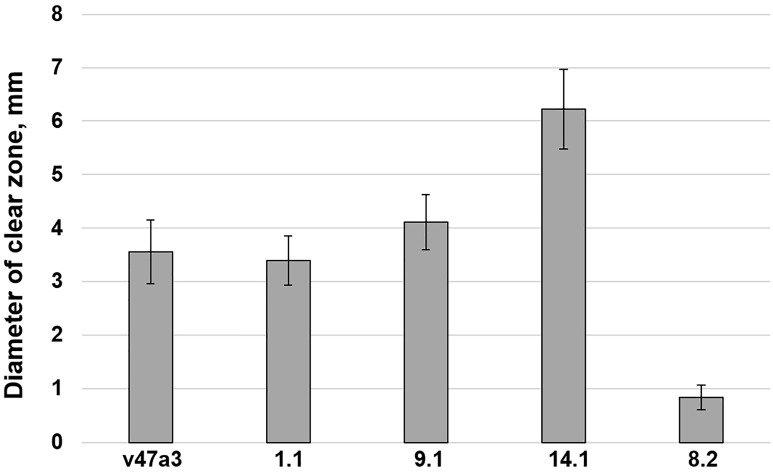
Assessment of the ability of clinical isolates of *C. albicans* to produce secretory proteinases. The diameters of the unstained amido black zones (clear zones) where BSA hydrolysis occurred around the fungal colonies were measured. Error bars represent a standard deviation (±SD) between nine technical replications.

Secretory and membrane-anchored aspartyl proteases (Saps) of *C. albicans* take part in fungal morphological switch, biofilm formation and the hydrolysis of diverse secreted host defense factors including AMPs [8]. We studied the ability of resistant clinical isolates of *C. albicans* to produce proteolytic enzymes by using BSA-containing agar plates. It was shown that clinical isolates v47a3, 1.1 and 14.1 effectively hydrolyzed BSA at pH 4.0, while strain 8.2 was almost unable to cleave this substrate (Figure 1; Supplementary Materials Figure S2).

**Figure 2:**
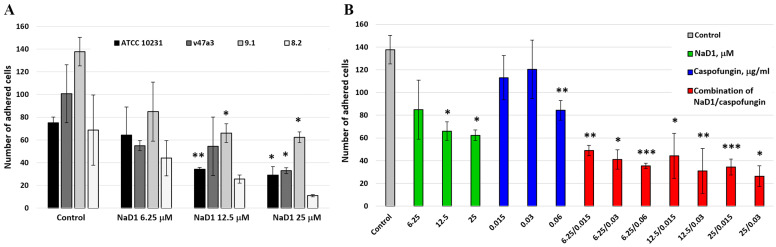
Adhesion of *C. albicans* cells to human Caco-2 epithelial monolayer. (**A**) Comparison of the ability of resistant strains ATCC 10231, v47a3, 9.1 and 8.2 to adhere to the Caco-2 monolayer and the influence of the tobacco defensin NaD1 at different concentrations on this process. (**B**) Inhibition of *C. albicans* 9.1 adhesion to epithelium by NaD1, caspofungin and their combination at various concentrations. Error bars represent a standard deviation (±SD) between three technical replications. Significance levels are * *p* ≤ 0.05, ** *p* < 0.01, *** *p* < 0.005. The numbers of adhered cells in untreated controls and samples treated by NaD1, caspofungin or their combinations were compared by unpaired two-sample *t*-test.

The adherence of *C. albicans* to human epithelial barriers is an essential first step of the infection process, which often results in the formation of biofilms [6]. We studied the ability of resistant strains ATCC 10231, v47a3, 9.1 and 8.2 to adhere to the apical membrane of the Caco-2 cell monolayer as a model of the intestinal epithelial barrier. It was demonstrated that all four fungal strains adhered to the Caco-2 monolayer with different adherence capacity. The highest attachment rate was shown for *C. albicans* 9.1 (27.5% versus 15, 20.1 and 13.7% for strains ATCC 10231, v47a3 and 8.2, respectively) (Figure 2A).

*C. albicans* forms heterogeneous and multilayered biofilms where fungal cells in both yeast-like and hyphae forms are present, surrounded by polymeric matrix. Biofilm formation takes place on the oral and vaginal mucosa and intestinal endothelium, as well as on dentures and various medical devices, including vascular and urinary catheters, and leads to a substantial reduction in the susceptibility of *C. albicans* to antifungal drugs [7]. Taking into consideration the diversity of environments in which biofilm formation can occur, we studied the ability of susceptible and resistant strains of *C. albicans* to form biofilms in Sabouraud broth and RPMI-1640 medium. We revealed that in nutrient-rich RPMI-1640, all *C. albicans* strains except the susceptible strain ATCC 18804 and clinical isolate v47a3 were able to form biofilms (Table 1; Supplementary Materials Figure S3). At the same time, resistant strains ATCC 10231 and 8.2 were also able to form biofilms in Sabouraud broth.

Thus, susceptible and resistant strains of *C. albicans* selected for the study of the anticandidal potential of the tobacco defensin NaD1 differed in their susceptibility to conventional antimycotics and their ability to transform from yeast-like to hyphal form in various nutrient media, produce secreted proteases, adhere to the epithelial barrier and form biofilms. However, our obtained data are limited due to the fact that we did not study the expression of virulence genes and toxin production by different strains of *C. albicans*, which may affect the effectiveness of the peptide in vivo experiments and should be further taken into account.

### 3.2. NaD1 Is Equally Effective Against Planktonic Cells of Both Susceptible and Resistant Strains of C. albicans

**Figure 3:**
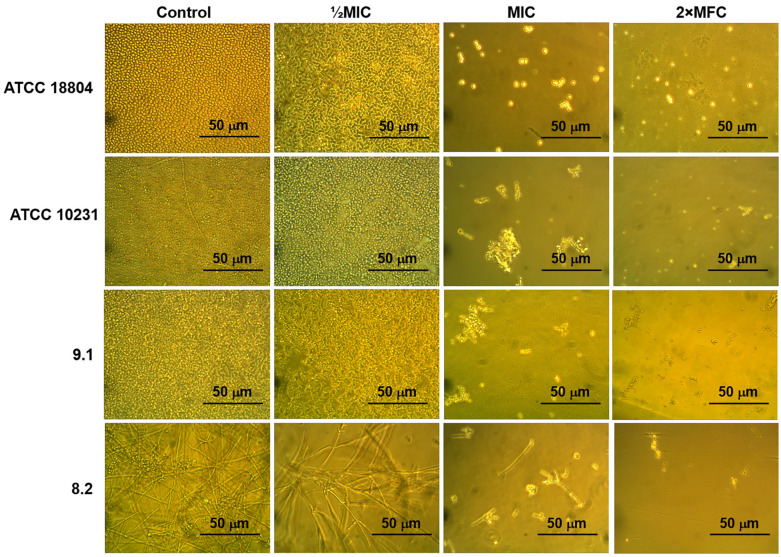
Effects of the tobacco defensin NaD1 at various concentrations on the growth of susceptible and resistant strains of *C. albicans* (400× magnification).

It is known that the tobacco defensin NaD1 possesses pronounced antifungal activity. The activity of this peptide against various collection and mutant strains of *C. albicans* has been shown in several studies [22,23]. Here, we performed a comparative study of the antifungal activity of NaD1 against susceptible and resistant to azoles and echinocandins strains of *C. albicans* described above. We showed that NaD1 was equally effective against all test microorganisms in Sabouraud broth including clinical isolate 8.2, which switched from a yeast-like to hyphal form in these conditions (Figure 3). The MIC value for all fungal strains was 6.25 µM (Table 2; Supplementary Materials Figure S4). In addition, NaD1 was shown to be fungicidal, and the MFC was 12.5 μM for all tested susceptible and resistant strains. Microscopic analysis revealed a decrease in cell number and also the occurrence of cell lysis at a peptide concentration of 25 μM (4× the MIC or 2× the MFC) in the case of all tested strains of *C. albicans* (Figure 3).

It was previously shown that the antifungal activity of NaD1, like a number of other AMPs, is significantly reduced in the presence of salts at physiological concentrations [23]. We showed the lack of inhibition of *C. albicans* ATCC 18804 growth in Sabouraud broth even at a concentration of 50 µM NaD1 in the presence of either 150 mM NaCl or 1.25 mM CaCl_2_ or 10% FBS. The activity of NaD1 less drastically decreased in the presence of 1.25 mM MgCl_2_, and an MIC of 12.5 µM and MFC of 25 µM were then observed (Supplementary Materials Table S3). The activity of NaD1 also decreased significantly in the nutrient-rich medium RPMI-1640, and MICs were above 50 µM in the case of all strains tested.

It was previously shown that NaD1 does not have pronounced hemolytic activity [23]. According to our data, NaD1 at a concentration of 50 µM caused lysis of about 2 or 10% of erythrocytes after incubation for 2 or 24 h, respectively. For comparison, the membrane-active peptide melittin from honeybee venom caused lysis of about 80 or 100% of erythrocytes at the same concentration (Supplementary Materials Figure S5). Recently, we showed that the tobacco defensin exhibited cytotoxic activity but at rather high concentrations [16]. A cell viability of approximately 90% and 70% was observed at the peptide concentration of 50 µM in the case of human peripheral blood mononuclear cells (PBMCs) and in the Caco-2 monolayer mimicking the gastrointestinal epithelial barrier, respectively. At the same time, melittin exhibiting nonspecific cytotoxic activity induced approximately 50% cell death at a concentration of 2.5 µM in both cases [16].

Thus, we have demonstrated that tobacco NaD1 was equally effective against all tested strains of *C. albicans.* This defensin was shown to be fungicidal and effectively inhibited growth of both susceptible and resistant to azoles and echinocandins strains of *C. albicans* in Sabouraud broth. At the same time, the antifungal activity of NaD1 was drastically reduced in the nutrient-rich RPMI-1640 medium.

### 3.3. NaD1 Shows Synergistic or Additive Effects Against Planktonic Cells of Susceptible and Resistant Strains of C. albicans with Antimycotics, Ebselen and Human Host Defense AMPs

The use of combination therapy, when antifungal compounds reinforce each other’s action, is a promising strategy to reduce the effective concentrations and, therefore, side effects of combined drugs. It was shown that some plant defensins can act synergistically with echinocandins, polyenes or inhibitors of bovine pancreatic trypsin against *C. albicans* [14]. In particular, NaD1 was demonstrated to have synergistic effects with caspofungin and inhibitors of bovine pancreatic trypsin [14].

Here, we studied the combined action of NaD1 against susceptible and resistant strains of *C. albicans* with: (1) antimycotics of three different classes—echinocandins (caspofungin, micafungin and anidulafungin), the azole derivative voriconazole and polyene AmB; (2) the synthetic organoselenium compound ebselen; (3) human host defense AMPs—cathelicidin LL-37 and β-defensin 2 (HBD2). All the abovementioned antimycotics are currently administered to treat various types of candidiasis in clinical practice. Ebselen exhibits a wide range of biological activities and has been shown to be effective in experimental models of vulvovaginal candidiasis [24]. LL-37 and HBD2 are produced by human epithelial and immune cells and possess pronounced anticandidal activity [25,26].

We showed that NaD1 acted synergistically with caspofungin against the susceptible strain ATCC 18804 and three resistant strains of *C. albicans* in Sabouraud broth. A minimal FICI value of 0.31 was found for strains 18804 and 9.1. At the same time, only additive action was observed in the case of resistant strains ATCC 10231 and 8.2 (Table 2; Supplementary Materials Figure S6). Minimal FICI values for combinations of NaD1 with other echinocandins, anidulafungin and micafungin, were higher and corresponded to the additive effects of the combined compounds (Table 3). Different effectiveness of combinations with various representatives of the echinocandin class has also been shown for other plant defensins [27]. It is worth noting that echinocandins are characterized by different structural organizations, effective concentrations, side effects and prevalence of resistant strains. At the same time, to the best of our knowledge, there are currently no data on the possible differences in the mechanism of their antifungal action [28].

Additive action against sensitive *C. albicans* strain ATCC 18804 was observed for the combination of NaD1 with fungistatic voriconazole (Table 3). Interesting effects were found for combinations of NaD1 with AmB. At high subminimal inhibitory concentrations (sub-MICs) of NaD1 and AmB, inhibition of antifungal activity was observed, while at lower concentrations, on the contrary, there was a mutual enhancement of their action in the case of both susceptible and resistant fungal strains (Supplementary Materials Figure S6). Minimal FICI value nevertheless corresponded to the additive action (Table 3). Apparently, at high concentrations, these compounds can form aggregates, which leads to a decrease in their antifungal activity. Ebselen, which inhibits the fungal plasma membrane H^+^-ATPase proton pump (Pma1p) [24], effectively inhibited the growth of both susceptible and resistant strains of *C. albicans* at a concentration of 12.5 µM and acted additively in combination with NaD1 (Table 3).

We demonstrated that human cathelicidin LL-37 acted equally effective against all strains used with an MIC of 12.5 µM. Synergistic effects of NaD1 and LL-37 were found in the case of three tested strains. The smallest FICI value, 0.38, was found in the cases of susceptible strain ATCC 18804 and clinical isolate 9.1. In the latter case, an FICI of 0.38 corresponded to two combinations, when NaD1 and LL-37 were present at 0.13× the MIC and 0.25× the MIC or 0.25× the MIC and 0.13× the MIC. This likely indicated a similar ability of NaD1 and LL-37 to enhance each other’s effects. Additive action of NaD1 and LL-37 was observed against some of the fungal strains tested (Table 2; Supplementary Materials Figure S6). HBD2 was also equally effective against all strains used with an MIC of 6.25 µM. HBD2 was also shown to act synergistically with NaD1 against three of the four fungal strains tested; however, this effect was less pronounced than in the case of LL-37. The lowest FICI value, 0.5, was demonstrated for combinations of NaD1 with HBD2 at 0.25× the MIC, which also indicated their equal contribution to the synergistic effect (Table 2).

Thus, we revealed the ability of the tobacco defensin NaD1 to act additively or synergistically with various compounds with different mechanisms of antifungal action. Additive effects against susceptible and resistant to azoles and echinocandins strains of *C. albicans* were found for the combination of NaD1 with ebselen and conventional antimycotics such as micafungin, anidulafungin, amphotericin B and voriconazole. Moreover, we showed for the first time that NaD1 acted synergistically not only with caspofungin but also with human host defense AMPs cathelicidin LL-37 and HBD2 against susceptible and resistant strains of *C. albicans.* We proposed that synergistic action of the tobacco defensin with LL-37 and HBD2, which is produced in fairly high concentrations by epithelial and immune cells during infection, may increase the effectiveness of NaD1 in vivo. At the same time, the lack of uniformity in the action of combinations of NaD1 with caspofungin, LL-37 and HBD2 was also demonstrated, since additive effects were only observed against some resistant fungal strains. We assumed that the different efficiency of the same combinations against various strains of *C. albicans* may be due to the variations in morphology, genetics and mechanisms of acquired resistance of the fungal strains.

### 3.4. Membrane-Disrupting Activity of NaD1 and Its Combinations with Caspofungin, LL-37 and HBD2

**Figure 4:**
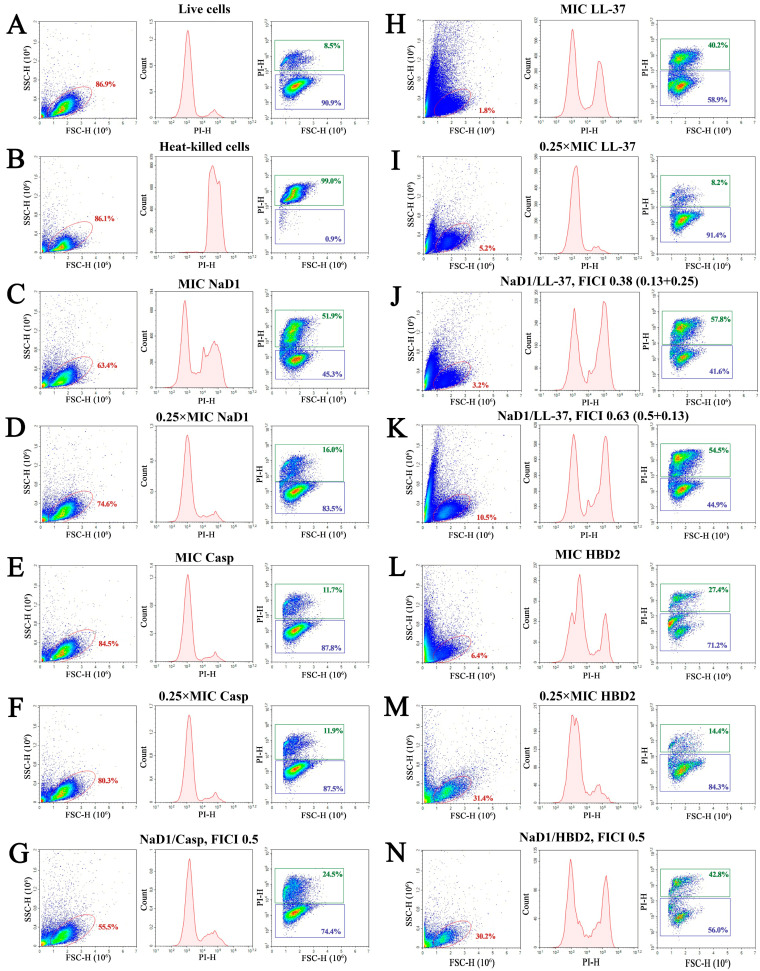
Flow cytometry analysis of viability of *C. albicans* ATCC 18804 cells after incubation for 2 h with NaD1 and its combinations with caspofungin (Casp), LL-37 or HBD2, measured by PI uptake. Live (**A**) and heat-killed (**B**) yeast-like cells were taken as negative and positive controls, respectively. Effects of NaD1 (**C**,**D**), caspofungin (**E**,**F**), LL-37 (**H**,**I**) and HBD2 (**L**,**M**) at the MIC (**C**,**E**,**H**,**L**) and 0.25× the MIC (**D**,**F**,**I**,**M**) are shown. Effects of combinations of NaD1 with caspofungin, LL-37 or HBD2 (**G**,**J**,**K**,**N**) on *C. albicans* viability are also demonstrated. Events on PI vs. count and FSC vs. PI plots are gated from the FSC vs. SSC diagram.

It is known that the mechanism of the antifungal action of NaD1 is characterized by complexity, but the main targets of this cationic peptide are components of the fungal cell membrane such as phosphatidylinositol-4,5-bisphosphate (PI(4,5)P2) and to a lesser extent phosphatidic acid (PA) [29,30]. The tobacco defensin affects the permeability of the plasma cell membrane interacting with these lipids [29,30]. Here, we used flow cytometry to study whether the observed synergistic effects of the combinations of NaD1 with caspofungin, LL-37 and HBD2 are determined by an increase in fungal membrane permeability. To this end, *C. albicans* ATCC 18804 cells were incubated with antifungal agent alone or in combination with NaD1 for 2 h and stained with fluorescent intercalating dye propidium iodide (PI), which can only penetrate dead cells with damaged membranes. Untreated and heat-killed fungal cells were used as negative and positive controls, respectively (Figure 4A,B).

We showed that NaD1 was able to damage the fungal membrane at the MIC but only a slight effect was observed at 0.25× the MIC after treatment for 2 h. In heat-treated or peptide-treated cells, the increase in PI fluorescence was manifested as a distinct shift in the peak along the x-axis yeast-like cells (Figure 4B–D, PI vs. count diagrams). PI-stained cells accounted for 52% in the case of NaD1 at MIC. Lysis of fungal cells took place in the case of NaD1 at 4× the MIC since a change in cell morphology was observed in the FSC vs. SSC diagram. This corresponded well with data from the microscopic analysis (Figure 3).

It is well known that human LL-37 also has a complex anticandidal action, and disruption of the fungal cell membrane is an important aspect of the killing mechanism of LL-37 [25]. In our experiments, LL-37 had a pronounced effect at the MIC, increasing the percentage of PI-stained cells to 40% (Figure 4H,I, PI vs. count diagrams), while in the case of 0.25× the MIC, the percentage of PI-stained cells in the corresponding gate was the same as in the control of live cells. It is worth noting that cell treatment with LL-37 resulted in a change in the cell morphology that can be associated with the lysis of *C. albicans* cells (Figure 4H,I, FSC vs. SSC diagrams). The percentage of PI-stained cells increased up to 58% in the presence of the synergistic combination of NaD1 and LL-37 with an FICI value of 0.38, where the concentration of the compounds was 0.13× the MIC and 0.25× the MIC, respectively (Figure 4J). Interestingly, a similar effect was observed for the additive combination of NaD1 and LL-37 with an FICI value of 0.63 where, contrariwise, LL-37 (0.13× the MIC) was taken in lower concentrations than NaD1 (0.5× the MIC) (Figure 4K). Thus, in the case of the combination of NaD1 and LL-37, we observed that they reinforce each other’s action on fungal membrane permeability, which can determine their synergistic action. It is known that NaD1 binds to β-glucan of the fungal cell wall, which affects its anticandidal action [29]. At the same time, LL-37 also binds to β-glucan as well as increases the activity of fungal Xog1p β-1,3-exoglucanase and reduces cell wall thickness [25]. Therefore, the synergistic effects of these peptides may also be due to the facilitation of NaD1 penetration through the fungal cell wall, but additional experiments are required to confirm this suggestion.

Human HBD2 has been shown to disrupt the permeability of the fungal membrane, probably in the same way as NaD1, by interacting with PI(4,5)P2, and it also inhibits the activity of fungal plasma membrane H^+^-ATPases [26,31]. In our study, HBD2 at MIC also increased membrane permeability but to a lesser extent than NaD1 or LL-37, and the percentage of PI-stained cells increased only to 27% (Figure 4L,M). At the same time, the percentage of PI-stained cells increased up to 43% in the presence of a synergistic combination of NaD1 and HBD2 with an FICI value of 0.5, when these compounds were taken at a concentration of 0.25× the MIC (Figure 4N). We proposed that the ability of both NaD1 and HBD2 to disrupt the integrity of the fungal cell membrane makes a significant contribution to their synergistic action.

It was suggested that the synergistic effects of plant defensin and echinocandins were related to the ability of the latter to inhibit the activity of (1,3)-β-D-glucan synthase and decrease 1,3-β-glucan levels in the fungal cell wall, facilitating the internalization of plant defensins, as well as increase the levels of target lipid PI(4,5)P2 in the fungal membrane [29]. Based on this suggestion, we hypothesized that synergistic effects of the combinations of NaD1 and caspofungin would be accompanied by an increase in membrane-disrupting activity of NaD1. But in our experiments, we did not receive confirmation of this hypothesis. Caspofungin at MIC did not affect membrane permeability after treatment for 2 h (Figure 4E,F). An increase in the percentage of PI-stained cells was almost not observed in the case of synergistic and additive combinations of NaD1 with caspofungin (shown in the example of the combination with an FICI value of 0.5, Figure 4G). The increase in the incubation time to 20 h led to an increase in membrane-disrupting activity of both NaD1 and caspofungin. After 20 h of incubation, NaD1 increased the percentage of PI-stained cells even at 0.25× the MIC (Supplementary Materials Figure S7B,C). Caspofungin had approximately the same effect at both 0.25× the MIC and the MIC. The percentage of PI-stained cells was about 38%, but a broader peak was observed in FSC vs. PI diagrams, probably indicating that various cell death pathways were activated (Supplementary Materials Figure S7D,E). A pronounced effect on membrane permeability was not observed in the case of synergistic and additive combinations of NaD1 and caspofungin (shown in the example of the combination with an FICI value of 0.5, Supplementary Materials Figure S7F). It is known that hyperproduction of reactive oxygen species (ROS) is another aspect of antifungal action of NaD1 [32]. Recent data showed that the antimicrobial effects of caspofungin are also associated with its ability to cause the accumulation of ROS in fungal as well as bacterial cells [33]. Moreover, new intracellular targets of caspofungin, such as histone deacetylase 4 (HDCA4), have recently also been proposed [34]. Therefore, we hypothesized that the synergistic effects of NaD1 and caspofungin may be determined by their intracellular action rather than a disruption in fungal membrane integrity. But further research is needed to confirm this assumption.

Thus, we demonstrated that NaD1 and human host defense AMPs such as cathelicidin LL-37 and defensin HBD2 are able to reinforce each other’s action on fungal membrane permeability, which could contribute to their synergistic effects against susceptible and resistant strains of *C. albicans*. At the same time, we did not observe an increase in the fungal membrane-disrupting action of NaD1 in combination with caspofungin and suggested that another mechanism of their synergistic anticandidal action, for example, one mediated by the intracellular targets of the combined substances, may occur.

### 3.5. NaD1 Inhibits the Adhesion of Resistant Strains of C. albicans to Human Epithelium

It is known that the ability of antifungal compounds to inhibit the adhesion of *C. albicans* cells to epithelial barriers may help to prevent fungal biofilm formation. It was shown that pea defensin Psd1 impaired *C. albicans* ability to adhere onto a poly-L-lysine (PLL)-coated glass [19]. Another plant defensin, gamma-hordothionin (D-lp1) from barley endosperm, inhibited the adherence of *C. auris* to buccal epithelial cells [35]. In our study, the ability of the tobacco defensin NaD1 at concentrations of 6.25, 12.5 and 25 μM to inhibit the adhesion of *C. albicans* cells to the human epithelial barrier was studied by using the Caco-2 monolayer and resistant fungal strains ATCC 10231, v47a3, 9.1 and 8.2. The study was conducted in nutrient-rich DMEM/F12 medium, used for culturing human epithelial-like cells, where NaD1 does not exhibit pronounced antifungal activity.

We demonstrated that NaD1 inhibited adhesion of all used strains at concentrations of 12.5 and 25 μM, but these data were not significant in the case of strain 8.2 (Figure 2A; Supplementary Materials Figure S8). *C. albicans* 9.1, which is characterized by the most pronounced ability to adhere, was chosen for further experiments. It was shown that NaD1 inhibited adhesion of this fungus by more than 50% at a concentration of 12.5, but its effects were insignificant at a concentration of 6.25 μM. Caspofungin also inhibited the adhesion of *C. albicans* 9.1 by approximately 40% at a concentration of 0.06 μg/mL, but had no significant effect at concentrations of 0.03 and 0.015 μg/mL. At the same time, more pronounced inhibition of fungal adhesion was observed with combinations of caspofungin and NaD1 at concentrations when they alone did not display significant effects. For example, inhibition by 70 and 64% was observed for combinations of NaD1 and caspofungin at concentrations of 6.25/0.03 and 6.25/0.015, respectively (Figure 2B; Supplementary Materials Figure S8). It is worth noting that NaD1 at a concentration of 25 μM exhibited a cytotoxic effect on the Caco-2 monolayer and cell viability of approximately 85% was observed [16]. Therefore, inhibition of *C. albicans* adhesion at this concentration may be associated not only with the effects of NaD1 on fungal cells but also on the human epithelial monolayer.

Thus, we showed that tobacco defensin NaD1 inhibits adhesion of resistant strains of *C. albicans* on the Caco-2 monolayer, mimicking the human intestinal epithelium, and enhances the anti-adherent properties of caspofungin.

### 3.6. NaD1 Inhibits the Formation of Biofilms by Resistant Strains of C. albicans and Enhances the Antibiofilm Activity of Caspofungin

It was shown that some plant defensins are able to prevent biofilm formation and increase the antibiofilm activity of caspofungin [14]. The ability to eradicate of mature biofilms of fungi of the *Candida* genus was shown for the salt-tolerant maize defensin ZmD32 [23]. In the case of NaD1, it was shown that this peptide does not eradicate the biofilms formed in RPMI-1640 [23]. Here, we studied the ability of the tobacco defensin alone and in combination with caspofungin to prevent biofilm formation by resistant strains of *C. albicans* in different conditions using Sabouraud broth and RPMI-1640. Resistant strains ATCC 10231 and 8.2 of *C. albicans*, which formed biofilms in both media, as well as resistant strain 9.1, which only formed biofilms in the RPMI-1640 medium, were chosen for these experiments based on the data described above (Table 1).

**Figure 5:**
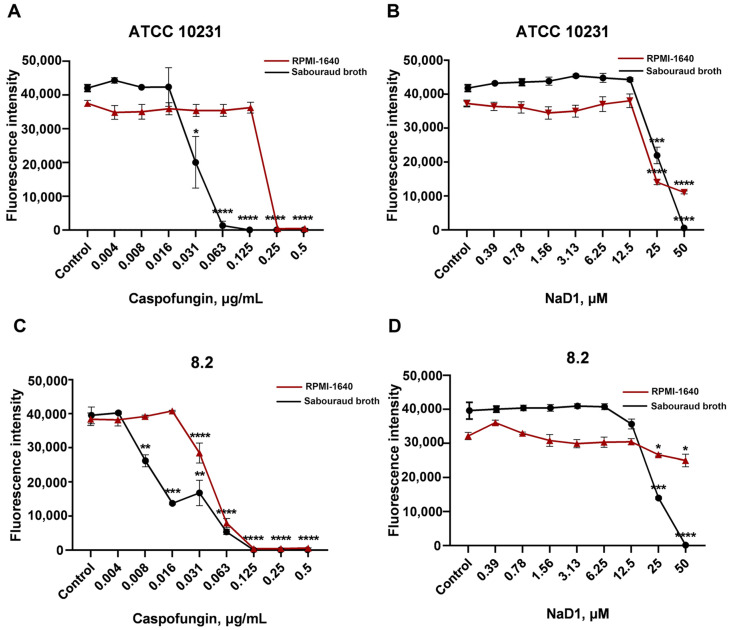
Effects of NaD1 (**B**,**D**) and caspofungin (**A**,**C**) on formation of biofilms by resistant strains ATCC 10231 (**A**,**B**) and 8.2 (**C**,**D**) of *C. albicans* in Sabouraud broth and RPMI-1640. Error bars represent a standard deviation (±SD) between three technical replications. Significance levels are * *p* ≤ 0.05, ** *p* < 0.01, *** *p* < 0.001, **** *p* < 0.0001. The untreated controls and samples treated by NaD1 or caspofungin were compared by unpaired two-sample *t*-test.

**Figure 6:**
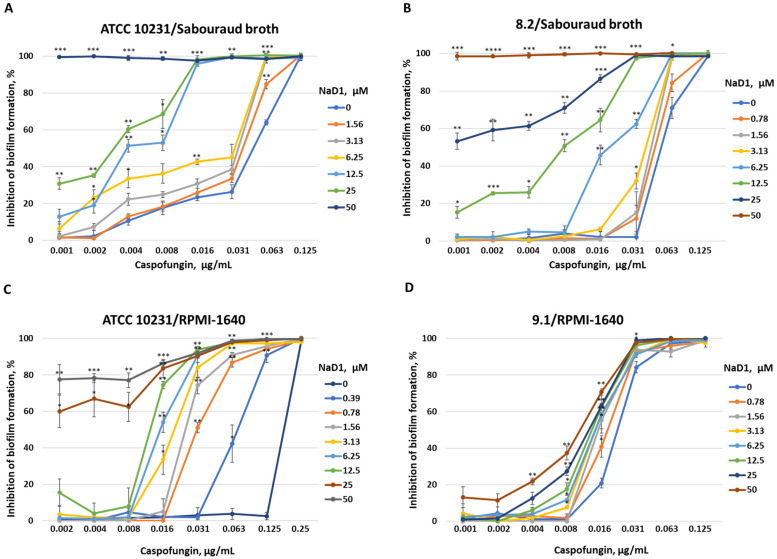
Effects of combinations of NaD1 with caspofungin on biofilm formation by resistant strains ATCC 10231 (**A**,**C**), 8.2 (**B**) and 9.1 (**D**) of *C. albicans* in Sabouraud broth (**A**,**B**) or RPMI-1640 (**C**,**D**). Error bars represent a standard deviation (±SD) between two technical replications. Significance levels are * *p* ≤ 0.05, ** *p* < 0.01, *** *p* < 0.001, **** *p* < 0.0001. The samples treated by caspofungin in different concentrations were compared with combinations of caspofungin in the same concentrations with NaD1 by unpaired two-sample *t*-test.

It was shown that NaD1 inhibited biofilm formation by both resistant strains in Sabouraud broth. The BIC, corresponding to 100% inhibition of biofilm formation, was 50 μM for both strains (Table 4, Figure 5). Synergistic effects of combinations of NaD1 and caspofungin on biofilm formation (BICI value of 0.5) were found for both resistant strains, ATCC 10231 and 8.2, in Sabouraud broth (Table 4, Figure 6). However, only additive effects were demonstrated in the case of planktonic cells of both strains for combinations of NaD1 and caspofungin (FICI value of 0.75) (Table 2). Previously, synergistic effects in relation to fungal biofilms, but not in relation to planktonic cells, were observed for combinations of some other plant defensins with caspofungin [36]. Yeast-like and hyphal forms present in biofilms of *C. albicans* differ from each other. The hyphae form has a thicker cell wall with increasing chitin and mannoprotein layers [4]. At the same time, β-glucans are less exposed components of the surface of fungal hyphae [4], which may possibly be the reason for the higher sensitivity of biofilms to combinations of NaD1 with caspofungin.

We demonstrated that NaD1 was much less active in RPMI-1640 against biofilms formed by resistant fungal strains ATCC 10231, 9.1 and 8.2, but inhibitory effects were still observed at high concentrations of the peptide (Table 4, Figure 5). A decrease in the antibiofilm activity of caspofungin in this nutrient-rich medium was also observed (Table 4, Figure 5). However, mutual enhancement of antibiofilm activity was observed for combinations of NaD1 with caspofungin in RPMI-1640, although the effects were different in the case of the three fungal strains used. The most pronounced effect was observed for the ATCC 10231 strain and the least pronounced for 8.2 (Table 4, Figure 6).

Thus, we showed that the tobacco defensin NaD1 inhibited biofilm formation by resistant strains of *C. albicans* and enhanced the antibiofilm activity of caspofungin. More pronounced effects were observed in Sabouraud broth, where a synergistic antibiofilm effect of NaD1 with caspofungin was found. In nutrient-rich RPMI-1640 medium, as expected, the effects of NaD1 and its combinations with caspofungin were less prominent, but an increase in antibiofilm activity of caspofungin by this defensin was still observed.

It is known that the antimicrobial activity of many AMPs, including human host defense AMPs, is significantly reduced in in vitro experiments in media with physiological salt content [37]. Despite this evidence, some of them demonstrate high efficacy in infection models in vivo [38]. In our study, we also observed that NaD1 exhibited much more pronounced antifungal properties in low-salt Sabouraud broth. However, even in nutrient-rich media, such as RPMI-1640 and DMEM/F12, tobacco defensin had an effect on resistant strains of *C. albicans*. In particular, NaD1 exhibited anti-adherent and antibiofilm effects and also enhanced caspofungin action. Recently, we showed that NaD1, like several other AMPs, exhibits immunomodulatory effects on various immune cells [16]. We proposed that the influence on the human immune system, as well as the ability of NaD1 to act synergistically with host defense AMPs LL-37 and HBD2, may contribute to the effectiveness of this peptide under infection in vivo. Nevertheless, the potency of NaD1 in vivo is difficult to predict, among other, due to its cytotoxic action in high concentrations and should be further tested in mouse models of candidiasis.

## 4. Conclusions

In this work, we investigated the antifungal potential of the tobacco defensin NaD1 against susceptible and resistant to azoles and echinocandins strains of *C. albicans*. We showed that NaD1 had the same fungicidal action and efficiency against all tested strains of *C. albicans* in Sabouraud broth. Using the checkerboard assay, we demonstrated that NaD1 had additive effects with ebselen and conventional antimycotics such as micafungin, anidulafungin, amphotericin B and voriconazole. Moreover, we showed for the first time that NaD1 was able to act synergistically not only with caspofungin but also with human host defense AMPs cathelicidin LL-37 and HBD2 against susceptible and resistant fungal strains. Using flow cytometry, we demonstrated that the ability of NaD1 and human host defense AMPs LL-37 or HBD2 to reinforce each other by enhancing membrane disruption could contribute to their synergistic anticandidal effects. Using the Caco-2 cell monolayer model, we showed that the tobacco defensin NaD1 was able to inhibit the adhesion of resistant strains of *C. albicans* on a human epithelial monolayer and enhance the anti-adherent properties of caspofungin. Finally, we demonstrated that NaD1 inhibited biofilm formation by resistant fungal strains and enhanced the antibiofilm effect of caspofungin in Sabouraud broth and less markedly in nutrient-rich RPMI-1640. To summarize all of our results, we suggest that the tobacco defensin NaD1 can be considered as a promising agent for the treatment of candidiasis, including that caused by resistant and multiresistant strains of *C. albicans.*

## Figures and Tables

**Table 1:** Characteristics of susceptible and resistant strains of *C. albicans* (collection strains ATCC 18804 and ATCC 10231; clinical isolates v47a3, 1.1, 9.1, 14.1 and 8.2).

*C. albicans*	Resistance to			Transition to Hyphal Form		Production of Proteases	Adhesion on Caco-2 Monolayer	Formation of Biofilms	
	Azoles	Echinocandins	Polyenes	Sabouraud Broth	RPMI-1640			Sabouraud Broth	RPMI-1640
ATCC 18804	−	−	−	−	−	nd	nd	−	−
ATCC 10231	+	−	−	−	+	nd	+	+	+
v47a3	+	−	−	−	−	+	+	nd	−
1.1	+	+ *	−	−	+	+	nd	nd	+
9.1	+	+ *	−	−	+	+	+	± ****	+
14.1	+	+ **	−	−	+	+	nd	nd	+
8.2	+	+ *	−	+	+	± ***	+	+	+

* Resistance only to anidulafungin; ** resistance to anidulafungin/micafungin; *** clear zone corresponding to BSA hydrolysis was less than 1 mm; **** fragile biofilms were formed, the destruction of which occurred during the washing procedure; nd—not determined.

**Table 2:** Activity of NaD1 and its combinations with caspofungin and human host defense AMPs LL-37 and HBD2 against planktonic cells of susceptible and resistant strains of *C. albicans* in Sabouraud broth.

*C. albicans*	NaD1, µM		MIC			Minimal FICI Values for Combinations of NaD1 and [A]/MIC_A_ + [B]/MIC_B_ * Values		
	MIC	MFC	Caspofungin, µg/mL	LL-37, µM	HBD2, µM	Caspofungin	LL-37	HBD2
ATCC 18804	6.25	12.5	0.04	12.5	6.25	**0.31** 0.06 + 0.25	**0.38** 0.13 + 0.25	**0.5** 0.25 + 0.25
ATCC 10231	6.25	12.5	0.04	12.5	6.25	0.75 0.25 + 0.5	**0.5** 0.25 + 0.25	**0.5** 0.25 + 0.25
v47a3	6.25	12.5	0.04	12.5	nd	nd	nd	nd
1.1	6.25	12.5	0.04	12.5	nd	**0.50** 0.25 + 0.25	0.75 0.5 + 0.25	nd
9.1	6.25	12.5	0.04	6.25	6.25	**0.31** 0.25 + 0.06	**0.38** 0.25 + 0.13 0.13 + 0.25	0.625 0.13 + 0.5
14.1	6.25	12.5	0.04	12.5	nd	**0.38** 0.13 + 0.25	0.56 0.5 + 0.06	nd
8.2	6.25	12.5	0.04	12.5	6.25	0.75 0.5 + 0.25	0.75 0.5 + 0.25 0.25 + 0.5	**0.5** 0.25 + 0.25

nd—not determined; MIC—minimal inhibitory concentration providing 100% inhibition of fungal growth; FICI—fractional inhibitory concentration index showing the type of action of combined compounds (synergism at FICI ≤ 0.5, additive action at 0.5 < FIC ≤ 1, independent action at 1 < FIC ≤ 2, antagonism at FICI > 2); FICI values corresponding to synergism are shown in bold; * [A]/[MICA]—ratio of NaD1 concentration in combination and its MIC, [B]/[MICB]—ratio of caspofungin, LL-37 or HBD2 concentrations in combinations and their MICs.

**Table 3:** Activity of combinations of NaD1 with conventional antimycotics of various classes and ebselen against planktonic cells of susceptible and resistant strains of *C. albicans* in Sabouraud broth.

Strain	MIC					Minimal FICI Values for Combination of NaD1 with				
	Anid, µg/mL	Micaf, µg/mL	Amph B, µg/mL	Voric, µg/mL	Ebselen, µM	Anid	Micaf	AmB	Voric	Ebselen
ATCC 18804	0.0025	0.04	0.156	0.015	12.5	0.516	1	0.507	0.75	0.516
ATCC 10231	0.0025	0.08	0.156	nd	12.5	1	0.516	0.507	nd	0.75

nd—not determined; Anid—anidulafungin; Micaf—micafungin; AmB—amphotericin B; Voric—voriconazole; MIC—minimal inhibitory concentration (50% inhibition of fungal growth for voriconazole and 100% growth inhibition for other compounds); FICI—fractional inhibitory concentration index showing the type of action of combined compounds (synergism at FICI ≤ 0.5, additive action at 0.5 < FIC ≤ 1, independent action at 1 < FIC ≤ 2, antagonism at FICI > 2).

**Table 4:** Inhibition of *C. albicans* biofilm formation in various media by NaD1, caspofungin and their combinations (concentrations of NaD1 and caspofungin are present in μM and μg/mL, respectively). Resistant strains ATCC 10231, 9.1 and 8.2 were used.

*C. albicans*		ATCC 10231	9.1	8.2
**Sabouraud Broth**				
NaD1	BIC_50_	25–50	nd	12.5–25
	BIC	50	nd	50
Caspofungin	BIC_50_	0.031–0.063	nd	0.008–0.063
	BIC	0.125	nd	0.125
NaD1/Caspofungin	BICI	0.5	nd	0.5
**RPMI-1640**				
NaD1	BIC_50_	12.5–25	>50	>50
	BIC	>50	>50	>50
Caspofungin	BIC_50_	0.125–0.25	0.016–0.031	0.031–0.063
	BIC	0.25	0.063	0.125
NaD1/Caspofungin	effect	increase in % inhibition of biofilm formation	a slight increase in % inhibition of biofilm formation	a slight increase in % inhibition of biofilm formation

nd—not determined; BICI—biofilm inhibitory concentration index showing the type of action of combined compounds (synergism at BICI ≤ 0.5, additive action at 0.5 < BIC ≤ 1, independent action at 1 < BIC ≤ 2, antagonism at BICI > 2).

## Data Availability

Data are contained within the article and Supplementary Materials.

## Supplementary Materials

The following supporting information can be downloaded at https://www.mdpi.com/article/10.3390/pathogens13121092/s1, Figure S1: Microscopic analysis of the ability of resistant clinical isolates of *C. albicans* to switch from yeast-like to hyphal form after cultivation for 4 h in various broths; Figure S2: Detection of proteases secreted by resistant clinical isolates of *C. albicans* using BSA-containing agar plates at pH 7.0 and 4.0; Figure S3: Comparison of the ability of resistant clinical isolates of *C. albicans* to form biofilms in RPMI-1640 medium; Figure S4: Antifungal activity of tobacco defensin NaD1 against different susceptible (A) and resistant strains (B) as well as resistant clinical isolates (C,D) of *C. albicans* in Sabouraud broth; Figure S5: Hemolytic activity of the tobacco defensin NaD1 after incubation for 2 (A) or 24 (B) h (hemoglobin release assay); Figure S6: Synergistic and additive effects of combinations of NaD1 with caspofungin (A,B), human cathelicidin LL-37 (C) and amphotericin B (AmB) (D) against susceptible and resistant strains of *C. albicans* ATCC 18804 and ATCC 10231; Figure S7: Flow cytometry analysis of viability of *C. albicans* ATCC 18804 cells after incubation for 20 h with NaD1 and its combination with caspofungin (Casp), measured by PI uptake; Figure S8: Influence of tobacco defensin NaD1, caspofungin and their combination on the ability of the clinical isolate of *C. albicans*, 9.1, to adhere onto the Caco-2 monolayer; Table S1: Susceptibility of collection strains and clinical isolates of *Candida albicans* to conventional antimycotics; Table S2: Characteristics of collection strains and clinical isolates of *C. albicans* used; Table S3: Effect of salts and fetal bovine serum (FBS) on the activity of NaD1 against *C. albicans* ATCC 18804.

## References

[1] World Health Organization WHO Fungal Priority Pathogens List to Guide Research, Development and Public Health Action. https://www.who.int/publications/i/item/9789240060241.

[2] Dadar M., Tiwari R., Karthik K., Chakraborty S., Shahali Y., Dhama K. (2018). *Candida albicans*—Biology, molecular characterization, pathogenicity, and advances in diagnosis and control—An update. Microb. Pathog..

[3] Gow N.A., van de Veerdonk F.L., Brown A.J., Netea M.G. (2011). *Candida albicans* morphogenesis and host defence: Discriminating invasion from colonization. Nat. Rev. Microbiol..

[4] Garcia-Rubio R., de Oliveira H.C., Rivera J., Trevijano-Contador N. (2020). The Fungal Cell Wall: *Candida*, *Cryptococcus*, and *Aspergillus* Species. Front. Microbiol..

[5] Lopes J.P., Lionakis M.S. (2022). Pathogenesis and virulence of Candida albicans. Virulence.

[6] Cheng S.C., Joosten L.A., Kullberg B.J., Netea M.G. (2012). Interplay between *Candida albicans* and the mammalian innate host defense. Infect Immun..

[7] Cavalheiro M., Teixeira M.C. (2018). *Candida* Biofilms: Threats, Challenges, and Promising Strategies. Front. Med..

[8] Kulshrestha A., Gupta P. (2023). Secreted aspartyl proteases family: A perspective review on the regulation of fungal pathogenesis. Future Microbiol..

[9] Morschhäuser J. (2002). The genetic basis of fluconazole resistance development in *Candida albicans*. Biochim. Biophys Acta..

[10] Carolus H., Pierson S., Lagrou K., van Dijck P. (2020). Amphotericin B and Other Polyenes-Discovery, Clinical Use, Mode of Action and Drug Resistance. J. Fungi.

[11] Beyda N.D., Lewis R.E., Garey K.W. (2012). Echinocandin resistance in Candida species: Mechanisms of reduced susceptibility and therapeutic approaches. Ann. Pharmacother..

[12] Cannon R.D., Lamping E., Holmes A.R., Niimi K., Baret P.V., Keniya M.V., Tanabe K., Niimi M., Goffeau A., Monk B.C. (2009). Efflux-mediated antifungal drug resistance. Clin. Microbiol. Rev..

[13] Finkina E.I., Ovchinnikova T.V. (2018). Plant defensins: Structure, functions, biosynthesis, and the role in the immune response. Russ. J. Bioorganic Chem..

[14] Finkina E.I., Shevchenko O.V., Fateeva S.I., Tagaev A.A., Ovchinnikova T.V. (2024). Antifungal Plant Defensins as an Alternative Tool to Combat Candidiasis. Plants.

[15] Manzanares P., Giner-Llorca M., Marcos J.F., Garrigues S. (2024). Fighting pathogenic yeasts with plant defensins and anti-fungal proteins from fungi. Appl. Microbiol. Biotechnol..

[16] Finkina E.I., Bogdanov I.V., Shevchenko O.V., Fateeva S.I., Ignatova A.A., Balandin S.V., Ovchinnikova T.V. (2024). Immunomodulatory Effects of the Tobacco Defensin NaD1. Antibiotics.

[17] Antoshina D.V., Balandin S.V., Finkina E.I., Bogdanov I.V., Eremchuk S.I., Kononova D.V., Kovrizhnykh A.A., Ovchinnikova T.V. (2024). Acidocin A and Acidocin 8912 Belong to a Distinct Subfamily of Class II Bacteriocins with a Broad Spectrum of Antimicrobial Activity. Int. J. Mol. Sci..

[18] Gonçalves S., Silva P.M., Felício M.R., de Medeiros L.N., Kurtenbach E., Santos N.C. (2017). Psd1 Effects on *Candida albicans* planktonic cells and biofilms. Front. Cell Infect. Microbiol..

[19] Moure M.C., Pérez Torrado R., Garmendia G., Vero S., Querol A., Alconada T., León Peláez Á. (2023). Characterization of kefir yeasts with antifungal capacity against *Aspergillus* species. Int. Microbiol..

[20] Sun J.N., Solis N.V., Phan Q.T., Bajwa J.S., Kashleva H., Thompson A., Liu Y., Dongari-Bagtzoglou A., Edgerton M., Filler S.G. (2010). Host cell invasion and virulence mediated by *Candida albicans* Ssa1. PLoS Pathog..

[21] Voropaev A.D., Yekaterinchev D.A., Urban Y.N., Zverev V.V., Nesvizhsky Y.V., Voropaeva E.A., Likhanskaya E.I., Afanasiev M.S., Afanasiev S.S. (2022). CDR1, CDR2, MDR1 and ERG11 expression in azole resistant *Candida albicans* isolated from HIV-infected patients in city of Moscow. Russ. J. Infect. Immun..

[22] Hayes B.M., Bleackley M.R., Wiltshire J.L., Anderson M.A., Traven A., van der Weerden N.L. (2013). Identification and mechanism of action of the plant defensin NaD1 as a new member of the antifungal drug arsenal against Candida albicans. Antimicrob. Agents Chemother..

[23] Kerenga B.K., McKenna J.A., Harvey P.J., Quimbar P., Garcia-Ceron D., Lay F.T., Shafee T.M.A., van der Weerden N.L., Hulett M.D., Craik D.J. (2019). Salt-tolerant antifungal and antibacterial activities of the corn defensin ZmD32. Front. Microbiol..

[24] Liang X., Pacuła-Miszewska A.J., Vartak R., Prajapati M., Zheng H., Zhao C., Mao G., Patel K., Fedosova N.U., Ścianowski J. (2024). N-3-Methylbutyl-benzisoselenazol-3(2H)-one Exerts Antifungal Activity In Vitro and in a Mouse Model of Vulvovaginal Candidiasis. Curr. Issues Mol. Biol..

[25] Memariani M., Memariani H. (2023). Antifungal properties of cathelicidin LL-37: Current knowledge and future research directions. World J. Microbiol. Biotechnol..

[26] Järvå M., Phan T.K., Lay F.T., Caria S., Kvansakul M., Hulett M.D. (2018). Human β-defensin 2 kills *Candida albicans* through phosphatidylinositol 4,5-bisphosphate-mediated membrane permeabilization. Sci. Adv..

[27] Cools T.L., Struyfs C., Drijfhout J.W., Kucharíková S., Lobo Romero C., van Dijck P., Ramada M.H.S., Bloch C., Cammue B.P.A., Thevissen K. (2017). A linear 19-mer plant defensin-derived peptide acts synergistically with caspofungin against *Candida albicans* biofilms. Front. Microbiol..

[28] Szymański M., Chmielewska S., Czyżewska U., Malinowska M., Tylicki A. (2022). Echinocandins—Structure, mechanism of action and use in antifungal therapy. J. Enzym. Inhib. Med. Chem..

[29] Bleackley M.R., Dawson C.S., Payne J.A., Harvey P.J., Rosengren K.J., Quimbar P., Garcia-Ceron D., Lowe R., Bulone V., van der Weerden N.L. (2019). The interaction with fungal cell wall polysaccharides determines the salt tolerance of antifungal plant defensins. Cell Surf..

[30] Payne J.A., Bleackley M.R., Lee T.H., Shafee T.M., Poon I.K., Hulett M.D., Aguilar M.I., van der Weerden N.L., Anderson M.A. (2016). The plant defensin NaD1 introduces membrane disorder through a specific interaction with the lipid, phosphatidylinositol 4,5 bisphosphate. Biochim. Biophys Acta.

[31] Andrés M.T., Fierro P., Antuña V., Fierro J.F. (2024). The Antimicrobial Activity of Human Defensins at Physiological Non-Permeabilizing Concentrations Is Caused by the Inhibition of the Plasma Membrane H+-ATPases. Int. J. Mol. Sci..

[32] Hayes B.M.E., Bleackley M.R., Anderson M.A., van der Weerden N.L. (2018). The Plant Defensin NaD1 Enters the Cytoplasm of *Candida Albicans* via Endocytosis. J. Fungi.

[33] Sumiyoshi M., Miyazaki T., Makau J.N., Mizuta S., Tanaka Y., Ishikawa T., Makimura K., Hirayama T., Takazono T., Saijo T. (2020). Novel and potent antimicrobial effects of caspofungin on drug-resistant *Candida* and bacteria. Sci. Rep..

[34] Aguiar T.K., Costa A.C., Neto N.A., Brito D.M., Freitas C.D., Neto J.M., Mesquita F.P., Souza P.F. (2024). Rise and fall of Caspofungin: The current status of Caspofungin as a treatment for *Cryptococcus neoformans* infection. Future Microbiol..

[35] Kamli M.R., Sabir J.S.M., Malik M.A., Ahmad A. (2022). Characterization of Defensin-like Protein 1 for Its Anti-Biofilm and Anti-Virulence Properties for the Development of Novel Antifungal Drug against *Candida auris*. J. Fungi.

[36] Vriens K., Cools T.L., Harvey P.J., Craik D.J., Braem A., Vleugels J., de Coninck B., Cammue B.P., Thevissen K. (2016). The radish defensins RsAFP1 and RsAFP2 act synergistically with caspofungin against *Candida albicans* biofilms. Peptides.

[37] Fathi F., Alizadeh B., Tabarzad M.V., Tabarzad M. (2024). Important structural features of antimicrobial peptides towards specific activity: Trends in the development of efficient therapeutics. Bioorganic Chem..

[38] Rodríguez-Castaño G.P., Rosenau F., Ständker L., Firacative C. (2023). Antimicrobial Peptides: Avant-Garde Antifungal Agents to Fight against Medically Important *Candida* Species. Pharmaceutics.

