# Supplementary material for: Effects of the Tobacco Defensin NaD1 Against Susceptible and Resistant Strains of Candida albicans
Source: Pathogens. 2024 Dec 10;13(12):1092. doi: 10.3390/pathogens13121092 (PMC11678012; doi:10.3390/pathogens13121092)
Supplement: Supplementary file 1 [file pathogens-13-01092-s001.zip › Supplementory materials.pdf]

Supplementary information for

Effects of the tobacco defensin NaD1 against sensitive and resistant strains of *Candida albicans*

Olga V. Shevchenko[1,2], Alexander D. Voropaev[3], Ivan V. Bogdanov[1], Tatiana V. Ovchinnikova[1,2], Ekaterina I. Finkina[1*]

[1] M.M. Shemyakin and Yu.A. Ovchinnikov Institute of Bioorganic Chemistry, Russian Academy of Sciences, 117997 Moscow, Russia; sh.o.v.2001@gmail.com (O.V.S.); contraton@mail.ru (I.V.B.); ovch@ibch.ru (T.V.O.); finkina@mail.ru (E.I.F.)
[2] Moscow Center for Advanced Studies, 123592 Moscow, Russia
[3]G.N. Gabrichevsky Research Institute for Epidemiology and Microbiology, 125212 Moscow, Russia; advoropaev@gmail.com (A.D.V.)
* Correspondence: finkina@mail.ru; Tel.: +7-495-335-0900

Table S1. Susceptibility of collection strains and clinical isolates of *Candida albicans* to conventional antimycotics (according to [S1]).

**Antifungal agent**	**MIC_{50} or MIC_{90} (mg/L)**						
	**ATCC 18804**	**ATCC 10231**	**v47a3**	**1.1**	**9.1**	**14.1**	**8.2**
Amphotericin B	0.5	0.5	0.5	1	1	0.5	0.5
Micafungin	0.015	0.015	0.008	0.015	0.015	**0.06**	0.015
Anidulafungin	≤0.015	0.03	0.03	**0.06**	**0.06**	**0.06**	**0.06**
Caspofungin	0.015	0.03	0.06	0.12	0.12	0.12	0.06
Fluconazole	0.5	**256**	**8**	**256**	**256**	**128**	**256**
Itraconazole	0.06	**16**	**0.12**	**16**	**16**	**8**	**>16**
Posaconazole	0.03	**8**	**0.12**	**8**	**8**	**4**	**>8**
Voriconazole	0.015	**8**	**0.5**	**8**	**8**	**4**	**>8**
Flucytosine	≤0.06	0.12	≤0.06	≤0.06	≤0.06	≤0.06	≤0.06

MIC – minimal inhibitory concentration (>90% inhibition of fungal growth for amphotericin B, but 50% growth inhibition for other compounds). MIC values corresponding to resistance to antifungal drugs are shown in bold.

Table S2. Characteristics of collection clinical isolates of *C. albicans* used (according to [S2]).

**Strain of *C. albicans***	**Overexpression of the gene**			
	ERG11	***MDR1***	***CDR1***	***CDR2***
v47a3	nd	nd	nd	nd
1.1	+	-	+	-
9.1	+	+	+	+
14.1	+	-	+	+
8.2	-	-	+	-

nd – not determined.

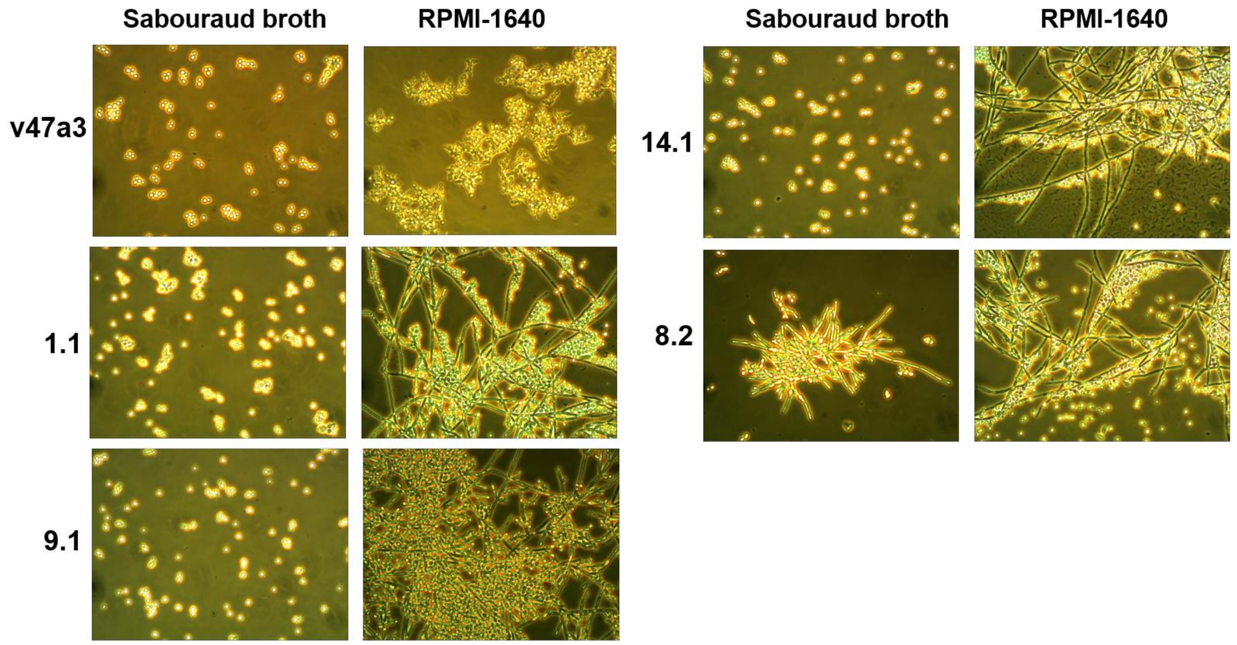

Figure S1. Microscopic analysis of the ability of resistant clinical isolates of *C. albicans* to switch from yeast-like to hyphal form after cultivation for 4 h in various broths (slides, magnification ×400).

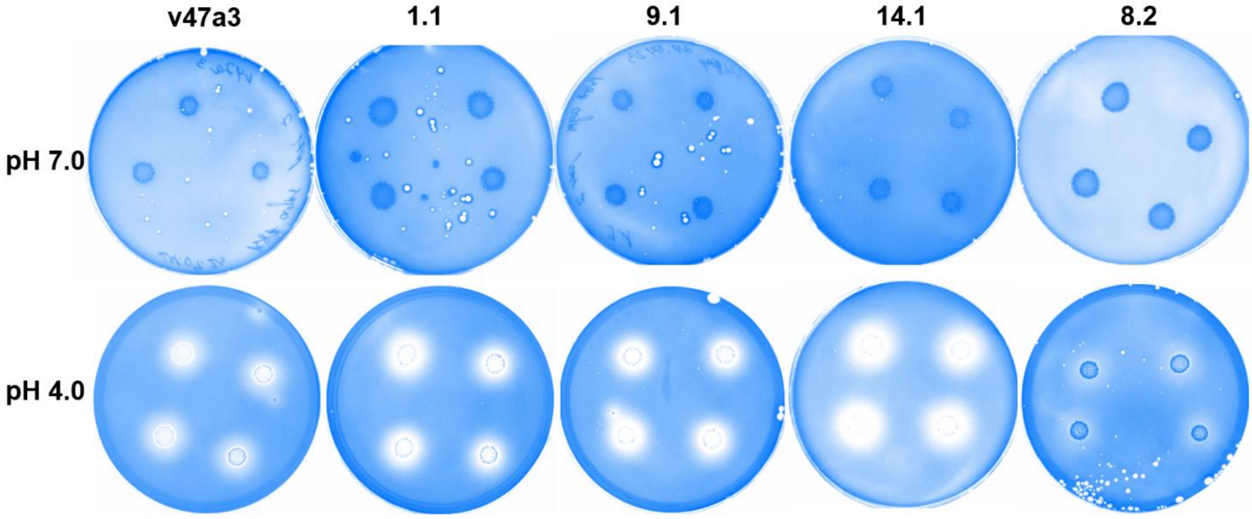

Figure S2. Detection of proteases secreted by resistant clinical isolates of *C. albicans* using BSA-containing agar plates at pH 7.0 and 4.0.

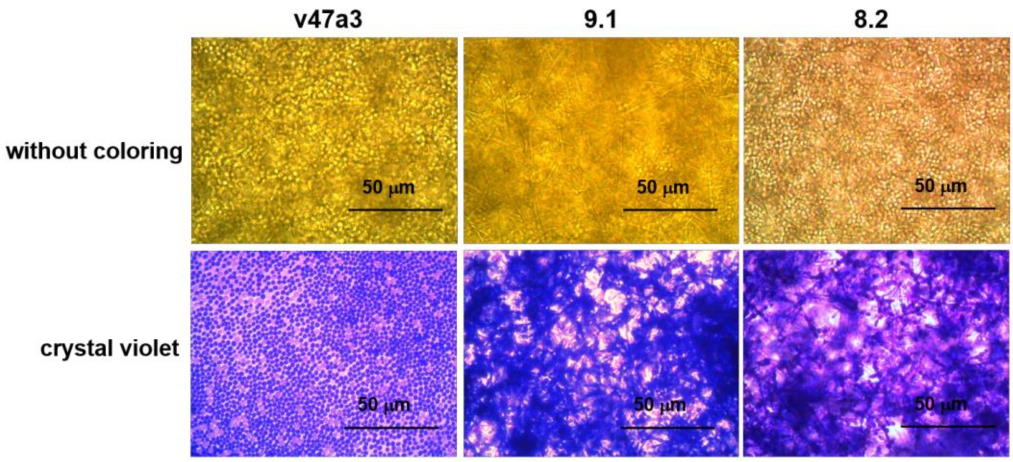

Figure S3. Comparison of the ability of resistant clinical isolates of *C. albicans* to form biofilms in RPMI-1640 medium (×400 magnification). Formed biofilms of 9.1 and 8.2 strains were stained with 0.1% crystal violet and washed with PBS. *C. albicans* v47a3 cells were stained without washing procedures.

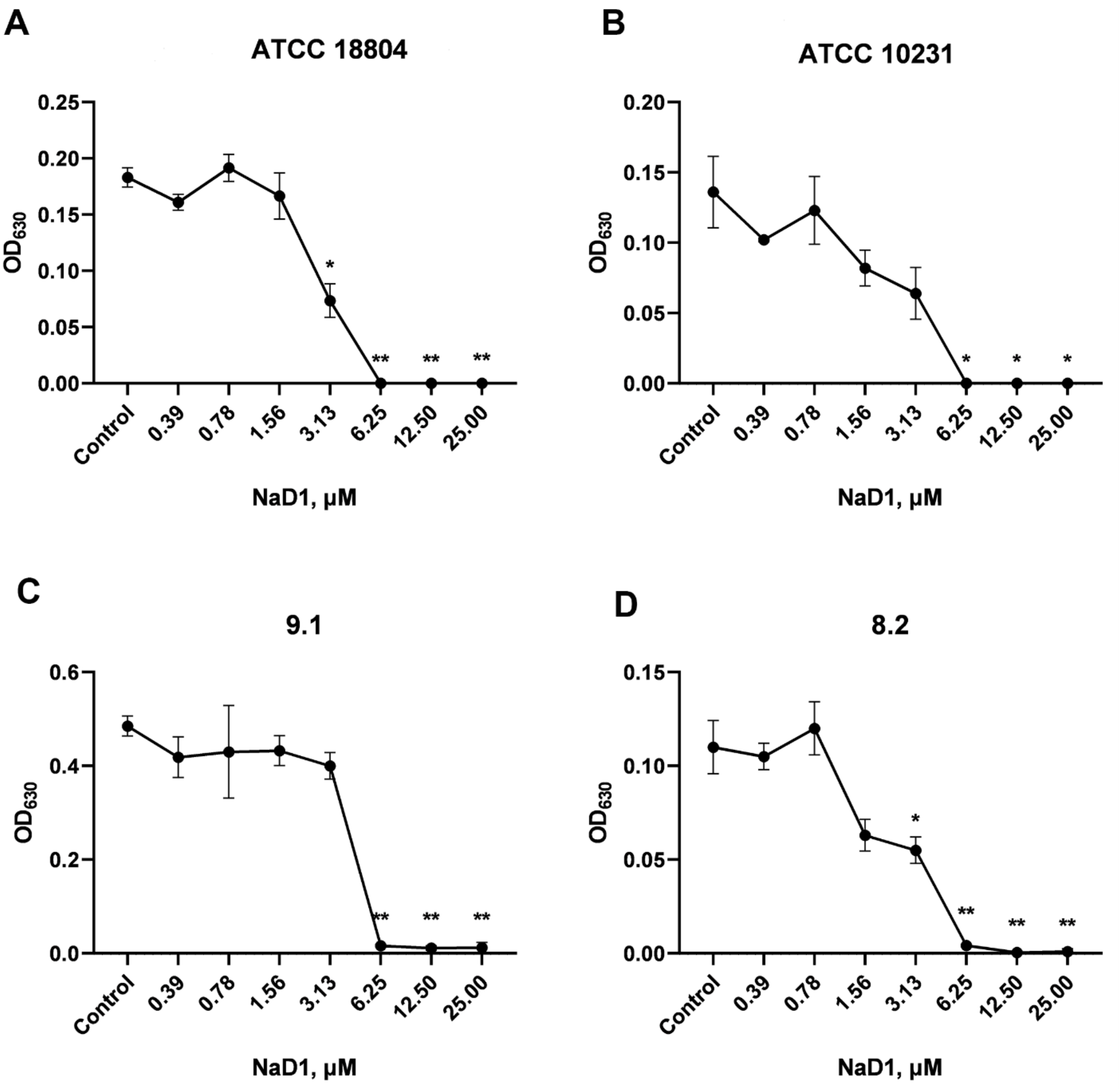

Figure S4. Antifungal activity of tobacco defensin NaD1 against different susceptible (A) and resistant strains (B) as well as resistant clinical isolates (C,D) of *C. albicans* in Sabouraud broth. Error bars represent a standard deviation (±SD) between technical replications. Significance levels are * $p < 0.05$, ** $p < 0.01$. The untreated controls and samples treated by NaD1 were compared by unpaired two-sample *t*-test.

Table S3. Effect of salts and fetal bovine serum (FBS) on the activity of NaD1 against *C. albicans* ATCC 18804 (concentrations of NaD1 are present in μM).

Peptide	No salts, no FBS		150 mM NaCl		1.25 mM CaCl2		1.25 mM MgCl2		10% FBS	
	MIC	MFC	MIC	MFC	MIC	MFC	MIC	MFC	MIC	MFC
NaD1	6.25	12.5	>50	nd	>50	nd	12.5	25	>50	nd

nd — not determined.

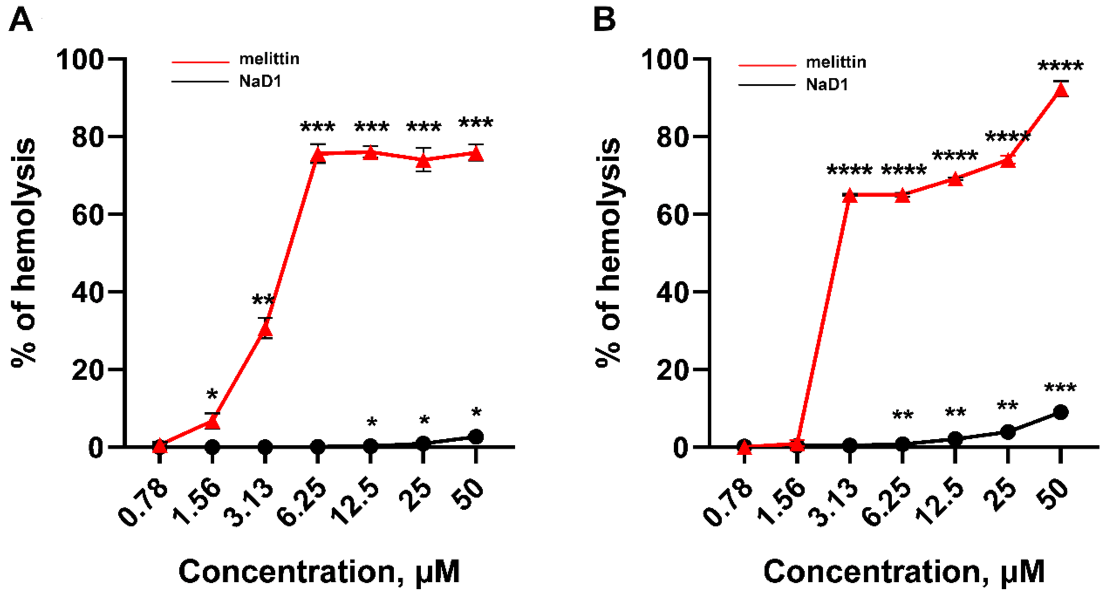

Figure S5. Hemolytic activity of the tobacco defensin NaD1 after incubation for 2 (A) or 24 (B) h (hemoglobin release assay). Membrane-active melittin from the venom of honeybees was used for comparation. Error bars represent a standard deviation (±SD) between technical replications. Significance levels are *$p \leq 0.05$, **$p < 0.01$, ***$p < 0.001$, ****$p < 0.0001$. Untreated control and samples treated by NaD1 or melittin were compared by unpaired two-sample *t*-test.

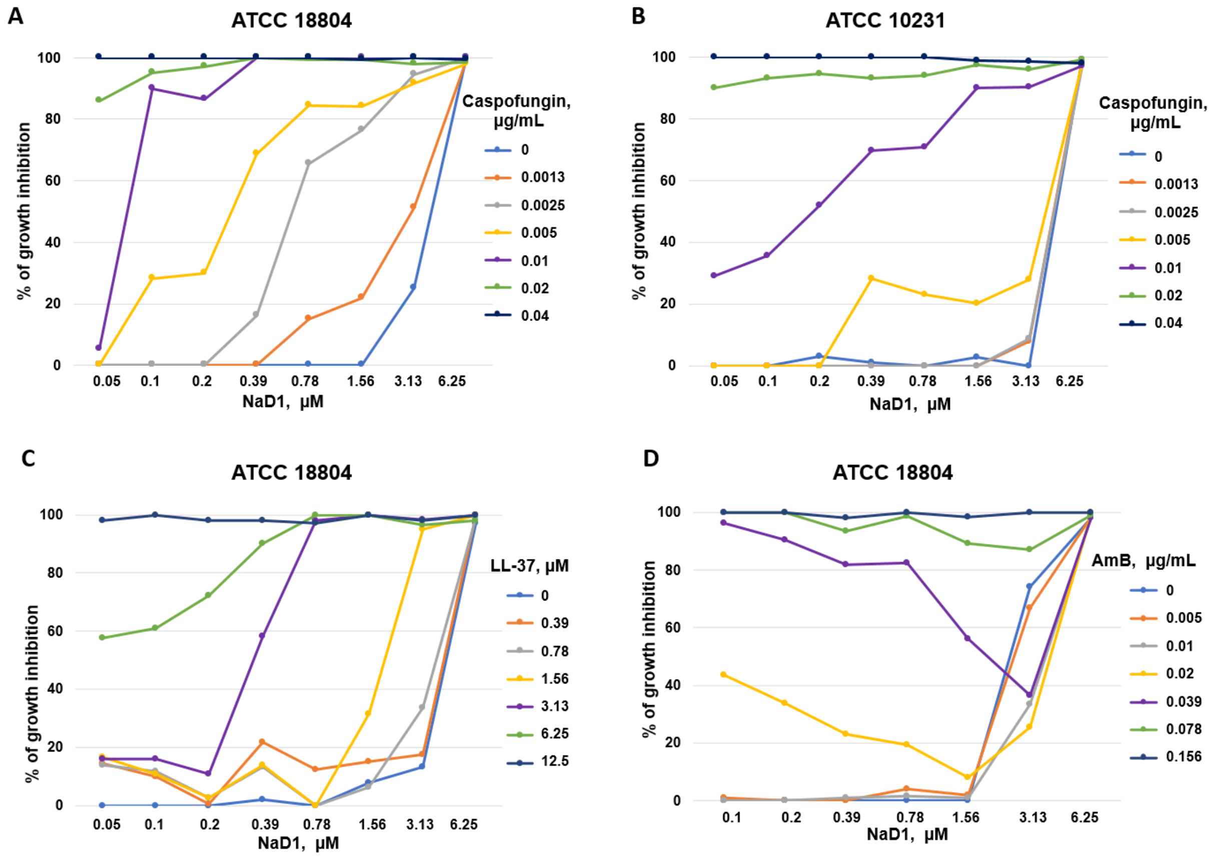

Figure S6. Synergistic and additive effects of combinations of NaD1 with caspofungin (A,B), human cathelicidin LL-37 (C) and amphotericin B (AmB) (D) against susceptible and resistant strains of *C. albicans* ATCC 18804 and ATCC 10231, respectively. Each graph shows data from a single checkerboard assay, the results of which were reproduced in three independent experiments.

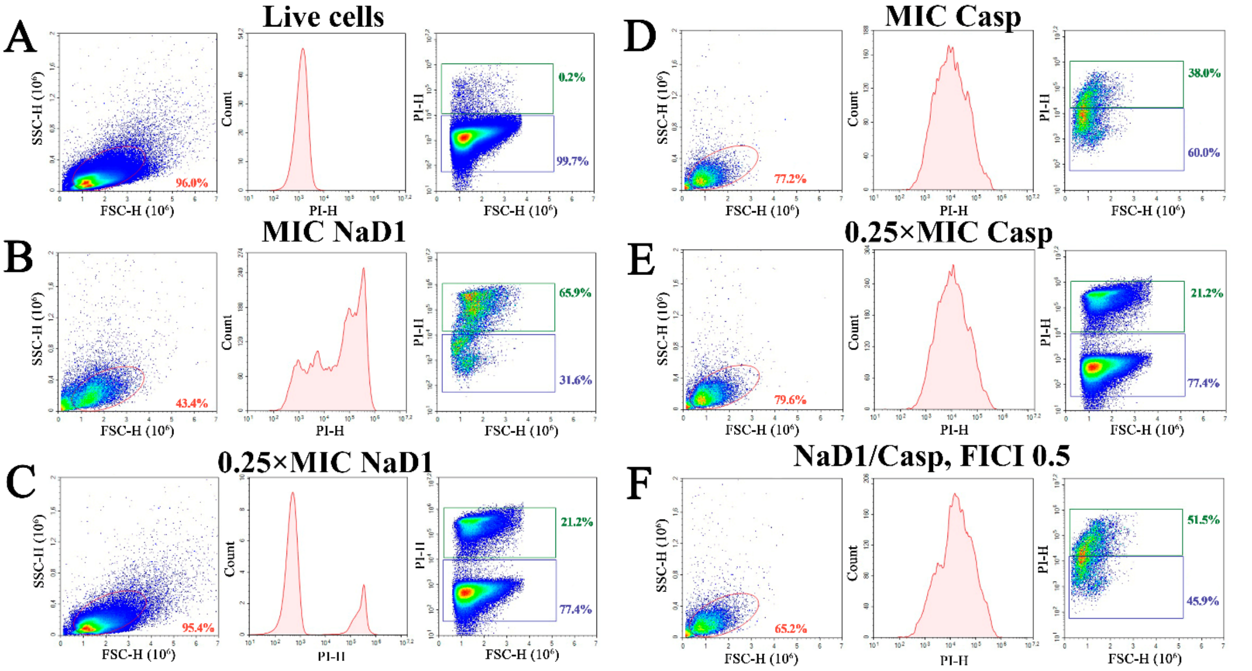

Figure S7. Flow cytometry analysis of viability of *C. albicans* ATCC 18804 cells after incubation for 20 h with NaD1 and its combination with caspofungin (Casp), measured by PI uptake. Live cells (A) were taken as negative control. Effects of NaD1 (B,C) and caspofungin (D,E) at concentrations of the MIC (B,D) and 0.25× the MIC (C,E) are showed. Effects of combination of NaD1 with caspofungin (F) on *C. albicans* viability are also demonstrated. Events on PI vs. count and FSC vs. PI plots are gated from FSC vs. SSC diagram.

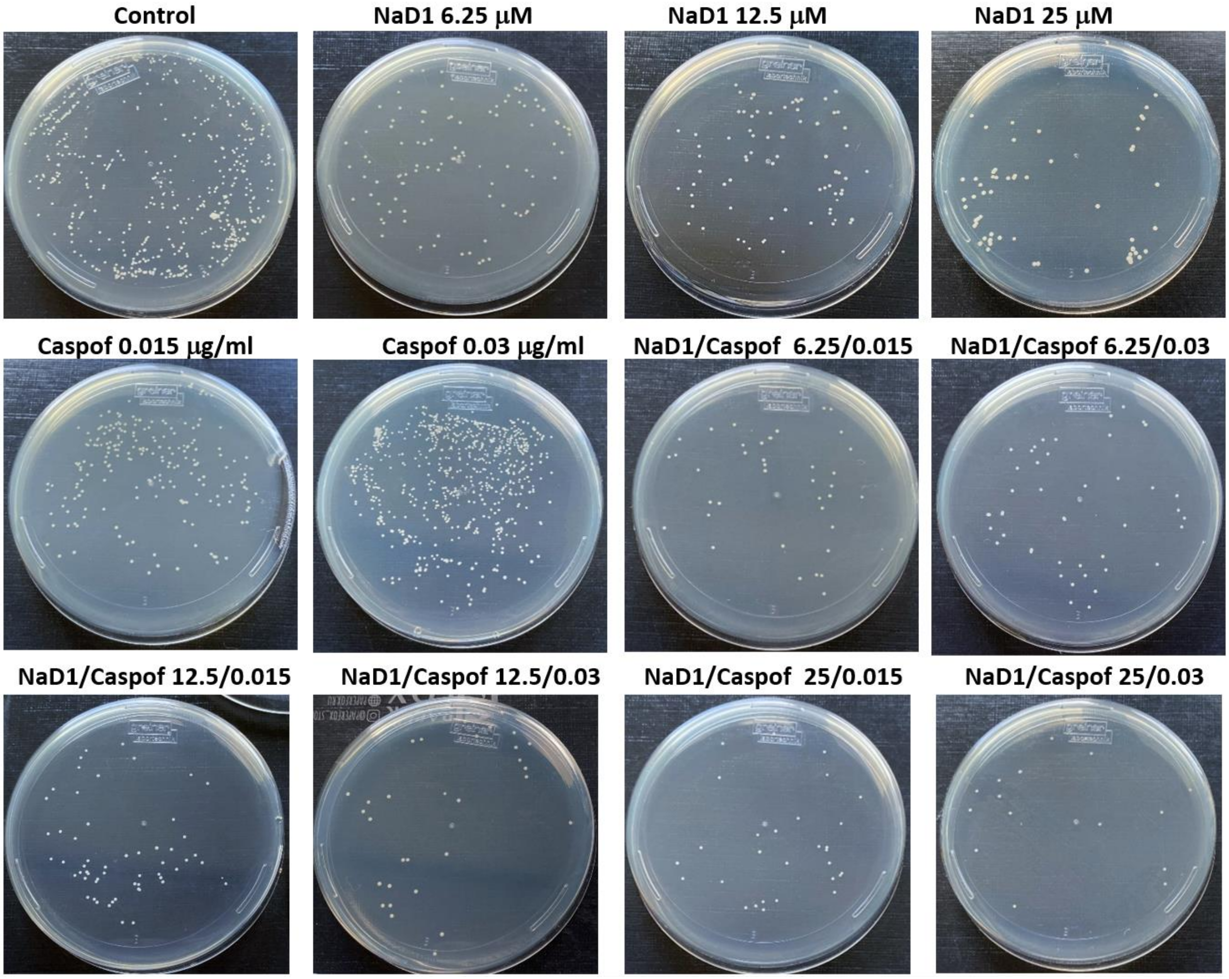

Figure S8. Influence of tobacco defensin NaD1, caspofungin and their combinations on the ability of the clinical isolate of *C. albicans* 9.1 to adhere onto the Caco-2 monolayer.

References

S1. EUCAST Definitive document E.DEF 7.3.2 Method for the determination of broth dilution minimum inhibitory concentrations of antifungal agents for yeasts. https://www.eucast.org/astoffungi/methodsinantifungalsusceptibilitytesting/susceptibility_testing_of_yeasts/

S2. Voropaev, A.D.; Yekaterinchev, D.A.; Urban, Yu.N.; Zverev, V.V.; Nesvizhsky, Yu.V.; Voropaeva E.A.; Likhanskaya, E.I.; Afanasiev, M.S.; Afanasiev, S.S. CDR1, CDR2, MDR1 and ERG11 expression in azole resistant *Candida albicans* isolated from HIV-infected patients in city of Moscow. *Russian Journal of Infection and Immunity*. **2022,** *12(5),* 929-937. doi: 10.15789/2220-7619-CCM-1931
